# Editorial: Insights in infectious agents and disease: 2022

**DOI:** 10.3389/fmicb.2024.1443636

**Published:** 2024-07-09

**Authors:** Axel Cloeckaert

**Affiliations:** INRAE, Université de Tours, UMR, ISP, Nouzilly, France

**Keywords:** viruses, bacteria, fungi, virulence, pathogenesis, pathology, antimicrobial resistance, epidemiology

## 1 Introduction

Major pandemics and epidemics, reviewed by Piret and Boivin (2021), involved bacterial infectious diseases such as plague, cholera or tuberculosis since past centuries, and viral infectious diseases such as flu and the most recent COVID-19 which emerged end of 2019, spread globally, and caused millions of deaths. Many infectious diseases leading to pandemics are caused by zoonotic pathogens, originating from an animal source (livestock, wildlife, or companion animals). In addition, some of them may be transmitted to humans through intermediate hosts such as arthropod hosts, which allow to group them also as vector-borne and zoonotic infectious diseases.

The present Research Topic, consisting of 40 published articles, provides an overview of recent developments in the field of bacterial, viral, and parasitic infectious diseases. This overview can be structured according to the type of infectious agent concerned, i.e. mainly bacterial, viral, or parasitic, and in the order of importance according to the number of articles published for each infectious agent type. It also reflects the current content of the section Infectious Agents and Disease of Frontiers in Microbiology.

## 2 Bacterial infections

The majority (29 out of 40) of articles published in this Research Topic concerned bacterial infectious diseases which can be further structured in five parts namely “Pathogenesis” including virulence factors, regulation of virulence, surface structures, and host response; “Antimicrobial resistance and therapy” including new developments in non-antibiotic treatment options such as phage therapy; “Epidemiology and bacterial diversity” concerning in particular risk factor and molecular epidemiology studies; “Diagnosis” with new developments in the field of pathogen detection and identification; and finally “Vaccine development” with the proposal of new vaccines formulations.

### 2.1 Pathogenesis

Pathogenesis-related articles published consisted of 11 Original Research and two Review articles, and covered the main pathogenic bacteria published in the section Infectious Agents and Disease such as Gram-positive bacterial species of the genera *Staphylococcus, Streptococcus*, and *Clostridium*; Gram-negative bacteria of the genera *Brucella, Escherichia, Francisella, Salmonella, Stenotrophomonas*, and *Vibrio*, with *Brucella, Francisella*, and *Salmonella* representing major intracellular pathogens; and finally *Mycobacterium tuberculosis*, which is also a major intracellular pathogenic species, with a particular cell wall distinct from Gram-positive or Gram-negative bacteria. Of related interest Soni et al. (2024) published a recent review on “understanding bacterial pathogenicity: a closer look at the journey of harmful microbes.” The authors classified bacterial pathogens based on Gram staining, as above, and precised that Gram-negative bacteria are more dangerous than their Gram-positive counterparts due to their formidable defenses. They possess an outer membrane that acts as a barrier, and efflux pumps that actively remove antibiotics. These bacteria can form biofilms, making them resistant to treatment, and can change surface structures to evade the immune system. In addition, what's particularly concerning is their propensity for multidrug resistance, rendering many antibiotics ineffective. The authors used also the bacterial classification based on their lifestyle, i.e. intracellular vs. extracellular as indicated above for the bacterial pathogens of the present Research Topic.

To start with Gram-positive extracellular pathogens, Afshar et al. reported biofilm formation and inflammatory potential of *Staphylococcus saccharolyticus* as a possible cause of orthopedic implant-associated infections. *S. saccharolyticus* is a coagulase-negative staphylococcal species and has some unusual characteristics for human-associated staphylococci, such as slow growth and its preference for anoxic culture conditions. This species is relatively abundant in the human skin microbiota, but its microbiological properties, as well as the pathogenic potential, have scarcely been investigated, despite being occasionally isolated from different types of infections including orthopedic implant-associated infections. Using several *in vitro* and cellular approaches including transcriptome analysis, the authors provided evidence for biofilm formation of this species together with a set of upregulated genes in biofilm-embedded cells, including factors involved in adhesion, colonization, and competition. In a cellular infection model *S. saccharolyticus* was in addition shown to trigger the secretion of pro-inflammatory cyto- and chemokines, but was less cytotoxic than *Staphylococcus aureus*. This study thus demonstrated a substantial pathogenic potential of the species *S. saccharolyticus*, which can be a potential cause of orthopedic implant-associated infections and other types of deep-seated infections. Deshmukh et al. studied gene expression of *S100a8/a9* as a potential biomarker to predict *S. aureus*-induced septic arthritis. Septic arthritis is an aggressive joint disease associated with high morbidity and mortality. Early antibiotic treatment is crucial for a better prognosis to save the patients from severe bone damage and later joint dysfunction. According to the authors, to date there are no specific predictive biomarkers for septic arthritis. By transcriptome sequencing analysis they identified *S100a8/a9* genes to be highly expressed in septic arthritis compared to non-septic arthritis at the early course of infection in an *S. aureus* septic arthritis mouse model. Regarding pathogenesis, importantly downregulation of *S100a8/a9* mRNA expression at the early course of infection was noticed in mice infected with the *S. aureus* Sortase A/B mutant strain totally lacking arthritogenic capacity compared with the mice infected with parental *S. aureus* arthritogenic strain. The authors concluded *S100a8/a9* gene expression may serve as a potential biomarker to predict septic arthritis, enabling the development of more effective treatment strategies. Lei et al. demonstrated that slipped-strand mispairing within a polycytidine tract in the transcriptional regulatory gene *mga* leads to M protein phase variation and Mga length polymorphism in Group A *Streptococcus*. The M surface protein is a major virulence factor of this group, and can be downregulated or lost during culture, due to a general mechanism called phase variation. This variation refers to a reversible on/off switch for control of expression of one or more proteins between individual cells of a clonal population. The authors thus identified slipped-strand mispairing within a polycytidine tract of the transcriptional regulatory gene *mga* as a reversible switch controlling M protein production phase variation in multiple common M types of Group A *Streptococcus*. Janžič et al. provided evidence for macrophage polarization during Group B *Streptococcus agalactiae* infection as being isolate specific, by studying a diverse set of strains belonging to different serotypes and sequence types on the immune response of THP-1 macrophages. Their data suggest that Group B isolates differ in their potential to become invasive or remain colonizing. In addition, colonizing isolates appeared to be more cytotoxic, whereas invasive isolates appeared to exploit macrophages to their advantage, avoiding the immune recognition and antibiotics. Schwanbeck et al. defined *Clostridioides difficile* minimal nutrient requirements for flagellar motility. As many gastro-intestinal pathogens, the majority of *Clostridioides difficile* strains express flagella together with a complete chemotaxis system. The resulting swimming motility is likely contributing to the colonization success of this important pathogen. The authors identified, by removal of individual amino acids from the medium, proline and cysteine as the most important amino acids that power swimming motility. A maximal and stable swimming motility was achieved with only four compounds, including the amino acids proline, cysteine and isoleucine together with a single, but interchangeable carbohydrate source. The authors expect that the identified “minimal motility medium” will be useful in future investigations on the flagellar motility and chemotactic behavior in *C. difficile*, particularly for the unambiguous identification of chemoattractants. Meurens et al. provided a review on *Clostridium botulinum* types C, D, C/D, and D/C. *C. botulinum* is the main causative agent of botulism, a neurological disease encountered in humans as well as animals. The different types are “toxinotypes” of nine types of botulinum neurotoxins. The A, B, and E types are the most frequently encountered in humans while the C, D, C/D, and D/C types are mostly affecting domestic and wild birds as well as cattle. The authors presented the current knowledge about toxinotypes C, D, C/D, and D/C in cattle and poultry with, amongst various other aspects, their epidemiological cycles. They also discussed the zoonotic potential of these toxinotypes and some possible ways of risk mitigation. An adapted and effective management of botulism outbreaks in livestock also requires a better understanding of these less common and known toxinotypes.

Regarding Gram-negative intracellular pathogens, Altamirano-Silva et al. studied phenotypes controlled by the *Brucella abortus* two component system BvrR/BvrS that appeared differentially impacted by BvrR phosphorylation. *B. abortus* is a zoonotic pathogen whose virulence depends on its ability to survive intracellularly at the endoplasmic reticulum derived compartment. The two-component system BvrR/BvrS (BvrRS) is essential for intracellular survival due to the transcriptional control of the type IV secretion system VirB and its transcriptional regulator VjbR. It is in addition a master regulator of several traits including membrane homeostasis by controlling gene expression of membrane components, such as Omp25. BvrR phosphorylation is related to DNA binding at target regions, thereby repressing or activating gene transcription. The BvrRS-contolled phenotypes investigated were resistance to polymyxins and intracellular survival. By using a set of mutants affecting BvrR phosphorylation, the authors provide evidence for a differential transcriptional response of genes depending on the phosphorylation status of BvrR, with resulting different phenotypes. The data suggest that unphosphorylated BvrR binds and impacts the expression of a subset of genes, as for example the dominant negative BvrR did not interact with the *omp25* promoter whereas it could interact with the *vjbR* promoter. BvrR thus appears to possess diverse strategies to exert transcriptional control on the genes it regulates and, consequently, impacting on the phenotypes controlled by this response regulator. Among pathogenic factors, biofilm production is known to enable bacteria to successfully colonize and persist in different environments and in different parts of the human body in the course of infection. Schaudinn et al. studied biofilm production in *Francisella tularensis* subspecies *holarctica* and showed that this subspecies is able to colonize natural aquatic *ex vivo* biofilms. Infections in humans are mostly associated with the highly virulent *F. tularensis* subspecies *tularensis* and the less virulent subspecies *holarctica*. Subspecies *holarctica* is more frequently associated with aquatic habitats. In the present sudy, the authors showed for the first time that a *F. tularensis* subspecies *holarctica* wild-type strain is able to successfully colonize an aquatic multi-species *ex vivo* biofilm. The authors speculate that subspecies *holarctica* might become more persistent in the environment when it forms its own biofilm or integrates in an existing one. Multi-species biofilms have been shown to be more resistant against stress compared to single-species biofilms. This may have an important impact on the long-term survival of *Francisella* in aquatic habitats and infection cycles in nature. Regarding biofilm formation, Zhang et al. studied its regulation in *Vibrio parahaemolyticus*, another important pathogen occurring in aquatic habitats and which is the primarily causative agent of the seafood-associated gastroenteritis. The authors provided evidence, using respective mutant backgrounds, that the quorum sensing regulatory proteins QsvR and OpaR coordinately repress biofilm formation by *V. parahaemolyticus*. More precisely, QsvR restored the biofilm-associated phenotypic changes caused by *opaR* mutation, and vice versa. In addition, QsvR and OpaR worked coordinately to regulate the transcription of bacterial colonization/biofilm formation genes such as exopolysaccharide-associated genes, type IV pili genes, capsular polysaccharide genes and c-di-GMP metabolism-related genes. Regarding virulence regulation, Hirakawa et al. provided evidence that the PapB/FocB family protein TosR acts as a positive regulator of flagellar expression and is required for optimal virulence of uropathogenic *Escherichia coli*. Uropathogenic *E. coli* (UPEC) is a major causative agent of urinary tract infections. The bacteria internalize into the uroepithelial cells, where aggregate and form microcolonies. UPEC fimbriae and flagella are important for the formation of microcolonies in uroepithelial cells. PapB/FocB family proteins are small DNA-binding transcriptional regulators consisting of ~100 amino acids that have been reported to regulate the expression of various fimbriae, including P, F1C, and type 1 fimbriae, and adhesins. In the present study, the authors showed that TosR, a member of this family, is a transcriptional activator that increases expression of the flagella *flhDC* operon genes, contributing to flagellar expression and optimal virulence. Ojiakor et al. investigated the evolutionary diversification of the *artAB* toxin locus of another important enterobacterial pathogen, namely *Salmonella enterica*. *S. enterica* is a diverse species of bacterial pathogens comprised of >2,500 serovars with variable host ranges and virulence properties. Accumulating evidence indicates that two AB5-type toxins, typhoid toxin and ArtAB toxin, contribute to the more severe virulence properties of the *Salmonella* strains that encode them. The authors focused on two genetic loci, *artAB* and *pltC*, the latter encoding an alternative delivery subunit for typhoid toxin. By assessing *Salmonella* genome sequences available in the NCBI genome database, they identified 7 subtypes of ArtAB toxins and 4 different PltC sequence groups that are distributed throughout the *Salmonella* genus. Interestingly, both *artAB* and *pltC* were located within numerous diverse prophages, indicating a central role for phages in their evolutionary diversification. This particular situation combined with genetic variation at both loci can thus be exploited by a continuously adapting pathogen such as *Salmonella* to yield novel toxins with distinct properties. Regarding pathogen evolution, Izydorczyk et al. studied the natural history and evolution of the opportunistic pathogen *Stenotrophomonas maltophilia* in the airways of adults with cystic fibrosis. Using genomic analyses, the authors investigated the natural history, transmission potential, and evolution of *S. maltophilia* in a large Canadian cohort of 321 cystic fibrosis patients over a 37-year period. These analyses suggested common, indirect sources as the origins of *S. maltophilia* infections in the clinic population. Infection within individual cystic fibrosis patients is driven by unique strains that are likely of environmental origins, as observed with other cystic fibrosis pathogens. While some patients may carry genetically related strains, these do not appear to be associated with patient-to-patient transmission but more likely with independent acquisition from environmental sources. The infection process is largely clonal at the SNP level, but significant diversity is present and driven by differences in gene content within strains.

To end this section on pathogenesis and pathogen evolution' collection of articles, more in relation to pathogen persistence and host-defense evasion, García-Bengoa et al. provided a review on the role of phagocyte extracellular traps during *Mycobacterium tuberculosis* infections and tuberculosis disease processes. *M. tuberculosis* infections remain one of the most significant causes of mortality worldwide. This pathogen is highly successful in evading the host-defense by manipulating host-signaling pathways. It concerns two cell types, macrophages and neutrophils. Both cells are known to act in multiple ways when encountering an invading pathogen, including phagocytosis, release of cytokines and chemokines, and oxidative burst. In addition, the formation of neutrophil extracellular traps (NETs) and macrophage extracellular traps (METs) has been described to contribute to *M. tuberculosis* infections. NETs/METs are extracellular DNA fibers with associated granule components, which are released upon activation of the cells by the pathogen or by pro-inflammatory mediators. In this review, the authors thus summarize the progress made in understanding the role of NETs/METs in the pathogenesis of tuberculosis.

### 2.2 Antimicrobial resistance and therapy

With the increasing rate over the past decades of multiple antibiotic resistance to almost all antimicrobial classes, carbapenems have been considered as a first choice to treat infections caused by multidrug-resistant bacteria. Cavallo et al. reported in a Review article a current view of the situation of *Acinetobacter baumanii* infection in the critically ill, with complex infections getting more complicated due to the increase of antimicrobial resistance and other persistence factors. Bacteremia, pneumonia, urinary tract, and skin and soft tissue infections are the most common presentations of *A. baumannii*, with attributable mortality rates approaching 35%. Due to the widespread prevalence of carbapenem-resistant *A. baumannii* (CRAB), colistin represents the main therapeutic option but high clinical failure rates have been reported as well for colistin monotherapy when used to treat CRAB infections. *A. baumannii* is also known to form biofilm on medical devices, including central venous catheters or endotracheal tubes. Thus, the worrisome spread of biofilm-producing strains in multidrug-resistant populations of *A. baumannii* poses a significant treatment challenge. The present review provided an updated account of antimicrobial resistance patterns and biofilm-mediated tolerance in *A. baumannii* infections with a special focus on fragile and critically ill patients. In relation with the topic above, Baek et al. reported gut microbiota alterations in critically Ill patients with carbapenem-resistant *Enterobacteriaceae* (CRE) colonization. The authors provided evidence that critically ill patients with CRE have a distinctive gut microbiota composition and community structure, altered short-chain fatty acid production and changes in the metabolic pathways. The authors suggested in addition further studies are needed to determine whether amino acids supplementation improves microbiota dysbiosis in patients with CRE. Paunkov et al. provided a proteomic analysis of metronidazole resistance in the human facultative pathogen *Bacteroides fragilis*. Most metronidazole-resistant *Bacteroides* isolates harbor *nim* genes, commonly believed to encode for nitroreductases which deactivate metronidazole. To assess proteomic changes following metronidazole resistance induction, the authors focused on a *B. fragilis* strain, either with (+) or without (–) the *nimA* gene. Among differentially expressed proteins in the *nim*A(+) strain, the flavodiiron protein FprA, an enzyme involved in oxygen scavenging, was identified. Interestingly, a far higher number of proteins were found to be differentially expressed in the *nimA*(–) strain upon metronidazole induction. They included factors for the import of hemin which were strongly downregulated, indicating impaired iron import in the *nimA*(–) strain. Together with other physiological data, the authors present a novel hypothetic model of metronidazole resistance and NimA function.

To combat or limit the spread of antimicrobial resistance in bacteria several approaches have been proposed or are under evaluation. Among them are new non-antibiotic strategies to limit or eradicate multi-resistant bacteria carriage without globally disrupting the microbiota. Bonnet et al. addressed in a Review article the question of decolonization of asymptomatic carriage of multidrug-resistant bacteria by bacteriophages. Asymptomatic colonization of the digestive tract by multidrug-resistant bacteria such as extended-spectrum beta-lactamase- or carbapenemase-producing *Enterobacterales*, poses several risks, including increased risk of infection, spread among patients and to the wider community, as well as resistance gene exchange between bacteria. This review discusses strategies for decolonizing the digestive tract of multidrug-resistant bacteria, including probiotics, fecal microbiota transplantation, and lytic bacteriophages. Highlighting that even though successful decolonization using lytic bacteriophages has been studied *in vivo* and *in vitro*, and observed clinically, limitations remain due to animal models, phage characteristics, and gut anatomy and further work is needed. In addition to this review, Li et al. reported that host CD3+ T-cells can significantly modulate phage treatment effects on bacterial bioburden in mouse models. Using *A. baumannii* phage mixtures the authors uncovered that the interplay between bacterial bioburden and host immune system may be bidirectional, and that there is an interaction between host CD3+ T-cells and phage dosage, which significantly impacts bacterial bioburden. Furthermore, the bacterial bioburden and wound size association was significantly modulated by the host CD3+ T-cells. This study provided evidence for strong relationships between host immune competency, therapeutic efficacy, bacterial clearance, and wound healing. Besides the obvious beneficial approaches of using bacteriophages to combat the spread of antimicrobial resistance, paradoxically Andersson et al. reported the enrichment of antibiotic resistance genes within bacteriophage populations in saliva samples from individuals undergoing oral antibiotic treatments. Indeed, horizontal gene transfer mediated through bacteriophages may also play an important role in the spread of antimicrobial resistance genes. In a cohort of Tanzanian patients suffering from bacterial infections, the authors demonstrated significant differences in the oral microbial diversity between infected and non-infected individuals, as well as before and after oral antibiotics treatment. Furthermore, the resistome carried both by bacteria and bacteriophages were shown to vary significantly, with extended cephalosporin *bla*_CTX − M−1_ resistance genes being mobilized and enriched within phage populations. Spread of resistance through bacteriophages in a biological context, as well in terms of treatment regimens should thus also be considered. As other approach to combat antimicrobial resistance, Troisi et al. provided a Mini Review article on a “A new dawn for monoclonal antibodies against antimicrobial resistant bacteria.” In almost 50 years since the introduction of the first technology that led to monoclonal antibody (mAb) discovery, enormous leaps forward have been made to identify and develop extremely potent human mAbs. While their usefulness has been extensively proved against viral pathogens, human mAbs have yet to find their space in treating and preventing infections from antimicrobial resistant bacteria and fully conquer the field of infectious diseases. The authors thus reviewed the novel and most innovative technologies that can support this goal and add powerful tools in the arsenal of weapons against resistant bacteria. Finally, Baquero et al. discussed in a Review article the natural detoxification of antibiotics in the environment in a one health perspective. The extended concept of one health integrates biological, geological, and chemical (bio-geo-chemical) components. Anthropogenic antibiotics are constantly and increasingly released into the soil and water environments. The fate of these drugs in the thin Earth space (“critical zone”) where the biosphere is placed determines the effect of antimicrobial agents on the microbiosphere, which can potentially alter the composition of the ecosystem and lead to the selection of antibiotic-resistant microorganisms including animal and human pathogens. In this review, all environmental aspects were carefully explored and discussed and the authors concluded the exploration of this complex field further requires a multidisciplinary effort in developing the molecular ecology of antibiotics, but could result in a much more precise determination of the one health hazards of antibiotic production and release.

### 2.3 Epidemiology and bacterial diversity

Epidemiology-related articles consisted of 1 Review and 3 Original Research articles. The review of Walton et al. focused not only on epidemiology but on all advances in cholera research from molecular biology to public health initiatives. The authors provided a complete picture on cholera disease and pathogenesis including evolutionary genomics, virulence, animal models, interaction with the gut microbiome, epidemiology, global distribution of cholera and at-risk populations, and cholera prevention initiatives. In the field of risk factors and risk analysis, Blumenröder et al. reported a cross-sectional pilot study on bacterial pathogens and maternal risk factors for neonatal infection in Sub-Saharan Africa. The study identified maternal urinary tract infection (UTI) and an elevated blood glucose level as potential maternal risk factors for early neonatal infection, an elevated blood glucose level, and maternal anemia for a late-onset infection. Simanjuntak et al. addressed the question of a suitable monitoring strategy for diarrhea risk assessment by a comparative pilot study on Gram-negative bacteria contaminating the hands of children living in urban and rural areas of Indonesia vs. Germany. Not surprising, fecal contamination correlated with hygienic conditions in the respective areas. The pilot study indicated that investigating hands of children for the prevalence of Gram-negative bacteria using selective media are a helpful method to monitor hygienic conditions, and thereby assess the risk for diarrhea-causing bacterial pathogens in the environment. Regarding pathogen's genetic diversity, Bauer et al. studied diversity of CRISPR-Cas type II-A systems in *Streptococcus anginosus*. Phylogenetic analysis supported the hypothesis that *S. anginosus* strains carry a variant CRISPR-Cas type II-A system relative to other streptococci. Bats constitute a reservoir of important zoonotic pathogens and Federici et al. provided an overview of bats microbiota and its implication in transmissible diseases. Bats, due to their being flying mammals and their increasing promiscuity with humans, have been recognized as hosts frequently capable of transmitting disease-causing microorganisms. Therefore, it is of considerable interest and importance to have a picture as clear as possible of the microorganisms that are hosted by bats. The authors reported on several pathogenic bacteria, including many carrying multidrug resistance, that are indeed common guests of these small mammals, underlining the importance of preserving their habitat, not only to protect them from anthropogenic activities, but also to minimize the spreading of infectious diseases.

### 2.4 Diagnosis

Regarding diagnosis and pathogen identification, Batool and Galloway-Peña provided a comprehensive review on clinical metagenomics, challenges and future prospects. Infections lacking precise diagnosis are often caused by a rare or uncharacterized pathogen, a combination of pathogens, or a known pathogen carrying undocumented or newly acquired genes. Thus, there is a need for an exhaustive and universal diagnostic strategy to reduce the fraction of undocumented infections. Compared to conventional diagnostics, metagenomic next-generation sequencing (mNGS) is a promising, culture-independent sequencing technology that is sensitive to detecting rare, novel, and unexpected pathogens with no preconception. In this review, the authors examined the current accomplishments, efficacy, and restrictions of mNGS in relation to conventional diagnostic methods and suggested potential approaches to enhance mNGS to its maximum capacity as a clinical diagnostic tool for identifying severe infections. Kakizaki et al. proposed a rapid identification method of bacteria using a multiplex polymerase chain reaction system for acute abdominal infections. Acute abdominal infections can be fatal if the causative organism(s) are misidentified. The multiplex PCR system used in this study showed a high detection rate for causative microorganisms in ascites and intraabdominal abscesses. This system may be suitable as an affordable rapid identification system for causative bacteria in these cases.

### 2.5 Vaccine development

In the field of vaccine development, Shattock et al. reported a self-amplifying RNA vaccine providing protection in a murine model of bubonic plague. The vaccine formula consisted of a combination of self-amplifying (sa) RNA constructs in lipid nanoparticles for the F1 and V antigens of *Yersinia pestis*. The data represent the first report of an RNA vaccine approach using self-amplifying technology and encoding both of the essential virulence antigens, providing efficacy against *Y. pestis*. This saRNA vaccine for plague has the potential for further development, particularly since its amplifying nature can induce immunity with less boosting. It is also amenable to rapid manufacture with simpler downstream processing than protein sub-units, enabling rapid deployment and surge manufacture during disease outbreaks.

## 3 Viral infections

### 3.1 SARS-CoV-2/COVID-19

The most important viral disease since the 20th century, namely COVID-19 disease, is caused by the severe acute respiratory syndrome coronavirus 2 (SARS-CoV-2). This coronavirus emerged end of 2019 and spread rapidly in human population all over the world becoming the first major pandemic of the 21th century, causing millions of deaths. Several variants have emerged in multiple successive waves during 3 years, leading health and governmental authorities to impose drastic prevention measures, such as lockdown. The development of several vaccines has helped to stop the spread and nowadays a global population immunity allows most people to live as in the situation before the COVID-19 outbreak. Nevertheless, this pandemic and associated research has greatly contributed to increase our knowledge regarding all aspects of a viral infectious disease, and to develop rapidly new diagnostic, therapeutic, prevention and vaccine strategies, with still interesting developments ongoing as can be seen in the articles of the present Research Topic.

Liu, Gan et al. reviewed therapeutic mechanisms and applications based on SARS-CoV-2 neutralizing antibodies. The authors provided an overview about antibodies targeting various regions [receptor-binding domain (RBD) regions, non-RBD regions, host cell targets, and cross-neutralizing antibodies], as well as the current scientific evidence for neutralizing-antibody-based treatments based on convalescent plasma therapy, intravenous immunoglobulin, monoclonal antibodies, and recombinant drugs. The functional evaluation of antibodies (i.e., *in vitro* or *in vivo* assays) was also discussed. Some current issues in the field of neutralizing-antibody-based therapies were also highlighted. Regarding vaccine development, Collett et al. reported the development of virus-like particles (VLPs) with inbuilt immunostimulatory properties as vaccine candidates. The development of VLP-based vaccines for human papillomavirus, hepatitis B and hepatitis E viruses represented a breakthrough in vaccine development. However, for dengue and COVID-19, technical complications, such as an incomplete understanding of the requirements for protective immunity, but also limitations in processes to manufacture VLP vaccines for enveloped viruses to large scale, have hampered VLP vaccine development. The authors described the development and characterization of novel VLP vaccine candidates using SARS-CoV-2 and dengue virus (DENV), containing the major viral structural proteins, as protypes for a novel approach to produce VLP vaccines. This unique vaccine formulation looks a promising, and much needed, new vaccine platform in the fight against infections caused by enveloped RNA viruses. Regarding vaccine-induced antibody responses, Puccini et al. studied kinetics of dried blood spot-measured anti-SARS-CoV2 Spike IgG in mRNA-vaccinated healthcare workers. The data of this study supported existing models showing that SARS-CoV2 vaccination elicits strong initial antibodies responses that decline with time but are transitorily increased by administering a vaccine booster. The authors also showed that using heterologous vaccine/booster combinations a stronger antibody response was elicited than utilizing a booster from the same vaccine manufacturer. Furthermore, by considering the impact of SARS-CoV2 infection occurrence in proximity to the scheduled booster administration, the authors confirmed that booster dose did not contribute significantly to elicit higher antibody responses. In the field of cellular pathogenesis, Garrett et al. studied niclosamide (NIC) as a chemical probe for analyzing SARS-CoV-2 modulation of host cell lipid metabolism. SARS-CoV-2 subverts host cell processes to facilitate rapid replication and dissemination, and this leads to pathological inflammation. NIC is a poorly soluble anti-helminth drug identified initially for repurposed treatment of COVID-19. This drug activates the cells' autophagic and lipophagic processes and the authors used it as a chemical probe to determine if it can modulate the host cell's total lipid profile that would otherwise be either amplified or reduced during SARS-CoV-2 infection. The authors observed that NIC treatment induced significant changes in host cell lipid metabolism that affected infectious virion production. Therefore, the authors posited that future screens of approved or new partner drugs should prioritize compounds that effectively counter SARS-CoV-2 subversion of lipid metabolism, thereby reducing virus replication, egress, and the subsequent regulation of key lipid mediators of pathological inflammation. Regarding virus persistence, Maffia-Bizzozero et al. reported the existence of viable SARS-CoV-2 Omicron sub-variants isolated from autopsy tissues. The authors investigated autopsy materials obtained from cadaveric donors and highlighted that SARS-CoV-2 can spread to multiple tissue locations such as the lungs, heart, liver, kidneys, and intestines, both after primary infection and after reinfections with the Omicron variant. In the field of therapy, Shapiro et al., in a Hypothesis and Theory article, raised the question regarding Anakinra authorized to treat severe coronavirus disease 2019; Sepsis breakthrough or time to reflect? Anakinra (recombinant human interleukin-1 receptor antagonist or rhIL-1ra) was approved by the European Medicines Agency (EMA) human medicines committee for use in COVID-19 disease that followed a prior indication extension 17 December 2021. The authors conducted a literature review and theoretical analysis of IL-1 blockade as a therapy to treat COVID-19. The author's analysis suggested Anakinra use as a COVID-19 therapy seems to rely on a view of pathogenesis that incorrectly reflects human disease. Since COVID-19 is an example of sepsis, COVID-19 benefit due to anti-inflammatory therapy contradicts an extensive history of unsuccessful clinical study. The authors suggested further experimentation is not a promising pathway to discover game-changing sepsis therapies and that a different kind of approach may be necessary.

### 3.2 Other viral infections

Long et al. investigated differences of gut microbiota between patients with negative and positive HBeAg in chronic hepatitis B and the effect of tenofovir alafenamide on intestinal flora. Hepatitis B virus (HBV) is the cause of severe liver diseases, such as liver fibrosis, cirrhosis, and liver cancer. This study revealed significant differences in gut microbiota composition and function between patients with HBeAg-positive and -negative chronic hepatitis B. Three other articles concerned animal and zoonotic viral diseases. Mazloum et al. provided a Review article on lumpy skin disease (LSD), a significant and emerging transboundary disease affecting cattle, buffaloes, and wild ruminants. As highlighted by the authors, LSD was initially confined to Africa and the Middle East but has recently spread across Eurasia, underscoring its previously underestimated impact. The causative agent, lumpy skin disease virus (LSDV), a poxvirus, was first identified in the 1940s in South Africa. LSDV spreads through indirect contact, shared water sources, and arthropods, complicating control measures. The virus evolves rapidly, generating new variants under diverse selective pressures. While primarily affecting livestock, certain wild ruminants are also susceptible, posing unknown risks to the disease's epidemiology. The review emphasizes the need for further research on these evolutionary dynamics and the impact on wild ruminant populations. Begeman et al. reported in a Hypothesis and Theory article the pathogenesis of zoonotic viral infections through lessons learned by studying reservoir hosts. Zoonotic viral infections that cause severe disease or even death in some people may be asymptomatic or mild in reservoir hosts. Comparison of the pathogenesis of these two host categories may potentially explain the difference in disease. The authors thus compared the pathogenesis of rabies virus, macacine alphaherpesvirus, West Nile virus, Puumala orthohantavirus, monkeypox virus, Lassa mammarenavirus, H5N1 highly pathogenic avian influenza, Marburg virus, Nipah virus, Middle East respiratory syndrome, and simian/human immunodeficiency viruses in both humans and reservoir hosts. Their study showed that most aspects of the pathogeneses were remarkably similar. The remaining differences lead to the identification of tipping points in the pathogeneses that are important for explaining the disease outcome in severe human cases. Further elucidating these tipping points by studying zoonotic viral infections in their reservoir hosts may teach us how to reduce the severity of zoonotic viral diseases in humans. Domanico et al. provided pathological and virological insights from an outbreak of European brown hare syndrome in the Italian hare (*Lepus corsicanus*). European brown hare syndrome (EBHS) is a highly contagious and fatal viral disease, mainly affecting European brown hares (*Lepus europaeus*). The etiological agent, EBHS virus (EBHSV), belongs to the *Lagovirus* genus within the *Caliciviridae* family. The molecular epidemiology and pathological investigation of this study supported previous reports of EBHS in *L. corsicanus* and further expanded the knowledge of the pathological and virological characteristics of the etiological agent. The ability of EBHSV to cause a fatal disease in the Italian hare represents a serious threat to the conservation of this vulnerable species, especially in populations kept in enclosed protected areas.

## 4 Parasitic infections

Liu, Yang et al. provided evidence that specific TLR-mediated HSP70 activation plays a potential role in host defense against the intestinal parasite *Giardia duodenalis*. *Giardia duodenalis*, an important flagellated noninvasive protozoan parasite, infects the upper small intestine and causes a disease termed giardiasis. Using cellular models of infection, the authors identified HSP70 as a potentially vital defender against *Giardia*, and revealed its correlation with specific TLR activation. The clinical importance of HSP70 has been extensively demonstrated, while its role as an effective therapeutic target in human giardiasis remains elusive and thus needs to be further clarified.

## Author contributions

AC: Writing – original draft, Writing – review & editing.

## References

[B1] Piret J. Boivin G. (2021). Pandemics throughout history. Front. Microbiol. 11:631736. 10.3389/fmicb.2020.631736 33584597 PMC7874133

[B2] Soni J. Sinha S. Pandey R. (2024). Understanding bacterial pathogenicity: a closer look at the journey of harmful microbes. Front. Microbiol. 15:1370818. 10.3389/fmicb.2024.1370818 38444801 PMC10912505

